# Temporal Asthma Patterns Using Repeated Questionnaires over 13 Years in a Large French Cohort of Women

**DOI:** 10.1371/journal.pone.0065090

**Published:** 2013-05-31

**Authors:** Margaux Sanchez, Jean Bousquet, Nicole Le Moual, Bénédicte Jacquemin, Françoise Clavel-Chapelon, Marc Humbert, Francine Kauffmann, Pascale Tubert-Bitter, Raphaëlle Varraso

**Affiliations:** 1 Inserm, CESP Centre for research in Epidemiology and Population Health, U1018, Respiratory and environmental epidemiology Team, F-94807, Villejuif, France; 2 Univ Paris-Sud, UMRS1018, F-94807 Villejuif, France; 3 University Hospital, Department of Respiratory Diseases, Hôpital Arnaud de Villeneuve, Montpellier, France; 4 Centre for Research in Environmental Epidemiology (CREAL), Barcelona, Spain; 5 Inserm, CESP Centre for research in Epidemiology and Population Health, U1018, Nutrition, hormones and women’s health Team, F-94807, Villejuif, France; 6 Assistance Publique Hôpitaux de Paris, DHU Thorax Innovation, Service de Pneumologie et Réanimation Respiratoire, Hôpital Bicêtre, Le Kremlin-Bicêtre, France; 7 INSERM U999, LabEX LERMIT, Centre Chirurgical Marie Lannelongue, F-92350, Le Plessis Robinson, France; 8 Univ Paris-Sud, Faculté de Médecine, F-94276, Kremlin-Bicêtre, France; 9 Inserm, CESP Centre for research in Epidemiology and Population Health, U1018, Biostatistics Team, F-94807, Villejuif, France; Universidade Federal do Acre (Federal University of Acre), Brazil

## Abstract

Variable expression is one aspect of the heterogeneity of asthma. We aimed to define a variable pattern, which is relevant in general health epidemiological cohorts. Our objectives were to assess whether: 1) asthma patterns defined using simple asthma questions through repeated measurements could reflect disease variability 2) these patterns may further be classified according to asthma severity/control. Among 70,428 French women, we used seven questionnaires (1992–2005) and a comprehensive reimbursement database (2004–2009) to define three reliable asthma patterns based on repeated positive answers to the ever asthma attack question: “never asthma” (n = 64,061); “inconsistent” (“yes” followed by “no”, n = 3,514); “consistent” (fully consistent positive answers, n = 2,853). The “Inconsistent” pattern was related to both long-term (childhood-onset asthma with remission in adulthood) and short-term (reported asthma attack in the last 12 months, associated with asthma medication) asthma variability, showing that repeated questions are relevant markers of the variable expression of asthma. Furthermore, in this pattern, the number of positive responses (1992–2005) predicted asthma drug consumption in subsequent years, a marker of disease severity. The “Inconsistent” pattern is a phenotype that may capture the variable expression of asthma. Repeated answers, even to a simple question, are too often neglected.

## Introduction

Disentangling asthma heterogeneity according to its temporal expression beyond accepted categories such as early childhood, childhood, adulthood, or very late onset of asthma is warranted [1]. While epidemiological observations from birth cohorts have provided important insights into both childhood-onset asthma and adulthood-onset asthma [2], [3], information regarding the variability of asthma expression in adulthood or over the whole life course remains scanty. In epidemiological studies, assessing the variability of asthma, a potential intrinsic characteristic of the disease, should help to clarify the response to environment and the determinants of asthma exacerbations [4]. Besides epidemiological respiratory studies, generally with few follow-ups during adulthood [5], large surveys on general health may include numerous follow-ups, sometimes over 20 years, but with limited information on asthma. We hypothesize that the in-depth analysis of repeated data on asthma, even if simple, may provide insight into asthma characterization.

There is an increasing interest in drug databases beyond drug surveillance. Information on dispensed drugs may allow asthma to be defined in the absence of other direct information [6]. Independent of subjects’ reports, they further provide interesting markers of disease activity, severity and control.

The E3N study (Etude Epidémiologique des Femmes de la Mutuelle Générale de l’Education Nationale) is a prospective study of major chronic diseases among members of the Mutuelle Générale de l’Education Nationale (MGEN), a French national health insurance plan. This study included approximately 100,000 women in 1990 [7]. Although based on a simple question, asthma was recorded throughout seven questionnaires, and associations with several risk factors have already been reported (body composition, dietary patterns, farming lifestyle, hormonal factors) [8]–[10].

Taking advantage of this very large epidemiological study, we address two aims: 1) to assess whether asthma patterns defined using simple asthma questions through repeated assessments could reflect disease variability; 2) to assess whether these patterns may be classified further, according to asthma severity and control. To address the aims, we combined the repeated information from 7 questionnaires on general health with dispensed drug data, and we analyzed the sequence of positive responses to asthma questions (inconsistent vs. consistent). We hypothesized that variable responses to asthma may capture the variable expression of the disease, and that the total number of positive answers to asthma may relate to subsequent drug consumption.

## Materials and Methods

### Study Design

The E3N study, initiated in 1990 (first questionnaire), included 98,995 women aged 40–65 years [7]. The 2^nd^ (1992), 3^rd^ (1993) and 4^th^ (1995) questionnaires were sent only to women who had answered the previous questionnaire. As from the 5^th^ questionnaire (1997), questionnaires were sent to all of the women who had responded to the first questionnaire (Figure S1 in File S1). No pulmonary function tests were performed. Questionnaires were self-completed and returned by mail. The E3N data are not publicly available.

### Setting

Two sources of information were used: 1) the main E3N study with sparse data on asthma but available for the whole E3N population from 1992 to 2005, 2) a dispensed drug database with objective data on asthma medications, but available only from 2004.

**Figure 1:**
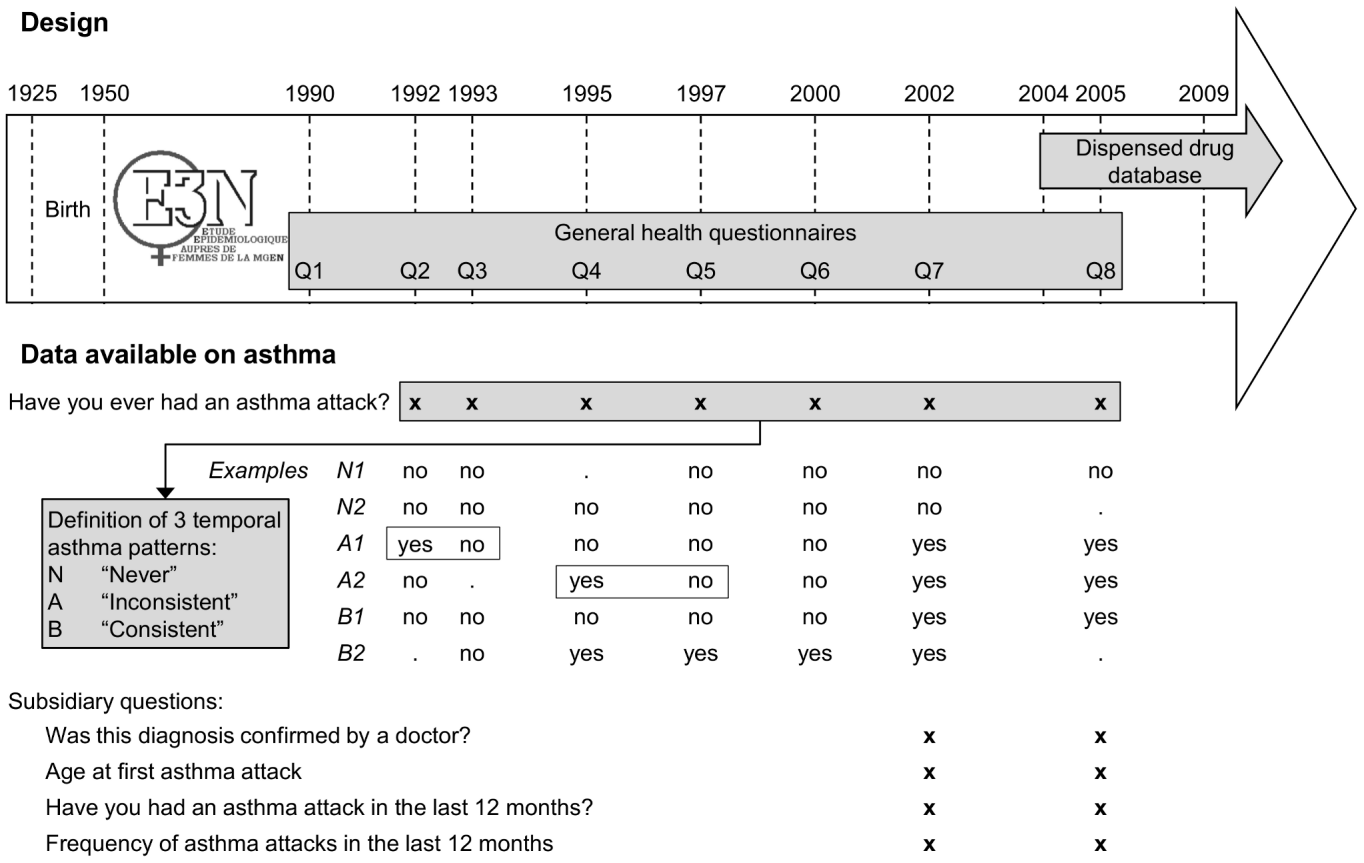
Study design and asthma assessment in the E3N cohort Study. Among the whole E3N population, three temporal asthma patterns were defined using 7 questionnaires sent between 1992 and 2005, all including a question on ever asthma. Four subsidiary questions were added in the 7^th^ and 8^th^ questionnaires (2002 and 2005). The dispensed drug database covered 6 complete years between 2004 and 2009. Two periods were taken into account in the analyses: 1) drug consumption in the 12 months preceding the exact return date of the 2005 questionnaire; 2) drug consumption between 2006 and 2009.

Main E3N study - All seven questionnaires from 1992 to 2005 included a simple question regarding asthma: “Have you ever had an asthma attack?”. Using these seven repeated answers, three temporal asthma patterns were defined (Figure 1 and Figure S1 in File S1). The first pattern was labeled “Never asthma”, due to the absence of positive answers over time. The second was labeled “Inconsistent answers” and included women with at least one “yes” to the asthma question, followed by a “no” in subsequent questionnaires. The third pattern, labeled “Consistent answers”, included women with fully consistent positive answers to the asthma question. The reliability of the three patterns was evaluated using standardized questions on asthma and the dispensed drug database (see File S1).

Dispensed drug database - The MGEN database contains comprehensive information on medications dispensed to all of the E3N women from 2004 onwards. We used data from 2004 to 2009 (Figure 1). Using the Anatomical Therapeutic Chemical (ATC) classification [11], we identified all dispensed asthma medications, in particular inhaled corticosteroids (ICS) and inhaled bronchodilators, as well as anticholinergics, as a typical chronic obstructive pulmonary disease (COPD) medication (see File S1).

### Participants

Our study population was composed of 70,428 women who returned the 8^th^ questionnaire in 2005 and who were still alive in 2009 (Figure S1 in File S1).

### Ethics

The study protocol was approved by the French Institutional Ethics Committee and all participants gave written informed consent. Ethical approval was granted to use the dispensed drug database.

### Variables and Data Measurements

From the main E3N study, besides the question on ever asthma asked between 1992 and 2005, 4 subsidiary questions were asked in 2002 and 2005 (7^th^ and 8^th^ questionnaires): “Was this diagnosis confirmed by a doctor?” (definition used in the American Thoracic Society standardization project [12]); “Age at first asthma attack”; “Have you had an asthma attack in the last 12 months?” and “Frequency of attacks in the last 12 months”. Childhood asthma was then defined as being less than 16 years old at the first asthma attack. In 2002, women were also asked about “Ever had an attack of shortness of breath at rest with wheezing”, allowing asthma to be defined according to the British Medical Research Council (BMRC) definition [13]. Body mass index (BMI), age and smoking status were investigated in 2005. Education level was investigated in 1990 and defined by the number of school years.

From the dispensed drug database, ICS were considered as both continuous and categorical variables (0 canisters, 1 to 3 canisters, 4 or more canisters dispensed). We considered 4 or more canisters of ICS dispensed during one year as a regular asthma controller medication [14]. Inhaled bronchodilators were considered as a continuous variable.

### Bias

To address potential information bias due to missing answers on asthma, we conducted a specific analysis of missing data (see File S1).

### Statistical Methods

The strategy of the analysis aimed at testing the hypothesis that “Inconsistent answers” reflect variability of asthma expression, both in the long term (childhood asthma with remission) and in the short term (asthma attack in the last 12 months). In order to assess the relationship between asthma answers and recent activity in the disease course, we analyzed, in the same 12-month period, dispensed drugs and answers regarding asthma attacks from the 2005 questionnaire. Finally, we tested the hypothesis that the total number of positive answers to asthma up until 2005 (maximum 6) may relate to subsequent drug consumption in the 2006–2009 period.

The statistical analysis used Chi-2, t student tests, analysis of variance and logistic regression to evaluate associations between qualitative and quantitative variables. Kappas to assess agreement were interpreted according to Landis and Kosh [15]. In this very large data set, the interpretation of the results took into account both the statistical significance and the clinically meaningful differences, as numerous associations were significant at the p<0.05 or p<0.001 level. All analyses were conducted using SAS, version 9 software (SAS Institute, Inc., Cary, North Carolina).

## Results

### Study Population

Among the 70,428 women, 64,061 (91.0%) were in the “Never asthma” pattern, 3,514 (5.0%) were in the “Inconsistent” pattern and 2,853 (4.0%) were in the “Consistent” pattern. The mean age was similar across the three patterns (Table 1). Women with “Consistent answers” were more often former smokers and had a higher mean BMI and a higher level of education, as compared to women in the “Never asthma” pattern. Women with “Consistent answers” had a greater mean BMI and a higher level of education than women with “Inconsistent answers”; no differences on smoking habits were found. The “Never asthma” pattern exhibited more missing asthma answers than the two other patterns. In both “Consistent” and “Inconsistent” patterns, more than 75% of the women fulfilled the BMRC and ATS definitions of asthma in 2002 and 2005 (Table 2).

**Table 1:** Characteristics of the population according to 1992–2005 temporal asthma patterns.

	1992–2005 temporal asthma patterns			p-value	
	“Never asthma” (1)	“Inconsistent answers” (2)	“Consistent answers” (3)	1 *vs.* 3	2 *vs.* 3
n	64,061	3,514	2,853		
Age in 2005 (years), mean (SD)	64.5 (6.4)	64.4 (6.4)	64.3 (6.3)	0.1	0.5
Smoking habits in 2005, %					
Never smokers	59.8	56.7	55.4	**	0.6
Former smokers	31.8	35.3	35.7		
Current smokers	6.2	5.6	6.3		
Missing	2.3	2.4	2.6		
Body mass index in 2005 (kg/m^2^), mean (SD)	23.9 (3.8)	24.5 (4.2)	24.8 (4.4)	**	*
Education number of school years, %					
≤11 years	11.6	10.8	12.4	**	*
12 to 14	49.2	48.2	45.2		
15 to 16	17.9	18.7	17.8		
≥17	17.1	18.8	20.1		
Missing	4.1	3.5	4.5		
Number of missing asthma answers from 1992 to 2005, %					
0	57.2	78.1	75.8	**	**
1	36.1	18.6	23.0		
2 or more	6.7	3.4	1.2		

p-value from χ^2^ test for categorical variable and from student test for continuous variable;

* p<0.05,

** p<0.001.

**Table 2:** Description of asthma according to 1992–2005 temporal asthma patterns.

	1992–2005 temporal asthma patterns		
	“Never Asthma”	“Inconsistent answers”	“Consistent answers”
**8^th^ questionnaire in 2005, n**	**64,061**	**3,514**	**2,853**
Ever had an asthma attack			
No	67.2	18.8	0.0
Yes	0.0	65.3	87.0‡
Missing data	32.8	15.9	13.0
*Among women with no missing data, n*	*43,063*	*2,954*	*2,481*
Asthma doctor diagnosis§		70.2	86.1**
Age at first asthma attack ≥16 years		44.3	60.9**
Asthma attack last 12 months		14.1	30.9**
**7^th^ questionnaire in 2002, n**	**61,833**	**3,412**	**2,720**
Ever had an asthma attack			
No	83.6	19.0	18.9
Yes	0.0	72.0	69.6
Missing data	16.4	9.0	11.4
*Among women with no missing data, n*	*51,695*	*3,104*	*2,409*
Asthma doctor diagnosis§		72.9	71.7
Age at first asthma attack ≥16 years		46.5	50.4**
Asthma attack last 12 months		14.2	27.9**
Ever had an attack of shortness of breath at rest with wheezing	0.8	45.0	41.5
Ever had an asthma attack or an attack of shortness of breathat rest with wheezing†	0.8	77.1	75.9*
Taking drugs for asthma (ever)		63.6	60.4

Data presented as %, unless otherwise stated. Questionnaires in 2002 and 2005 were sent to the whole E3N population.

* p<0.05.

** p<0.001 from χ^2^ test “Inconsistent” pattern vs. “Consistent” pattern;

§ American Thoracic Society definition of asthma;

† British Medical Research Council definition of asthma;

‡ Out of whom 841 (33.9%) responded positively for the first time in the 2005 questionnaire, i.e. incident asthma in 2005.

### Variability of Asthma Expression

Compared to women from the “Consistent” pattern, women from the “Inconsistent” pattern reported childhood-onset asthma more often (crude odds ratios [95% confidence interval] were 1.47 [1.28–1.69], 1.74 [1.51–1.99] in 2002, 2005 respectively) and less asthma attacks in the last 12 months (0.41 [0.36–0.48], 0.32 [0.28–0.37] in 2002, 2005 respectively) (Table 2).

**Figure 2:**
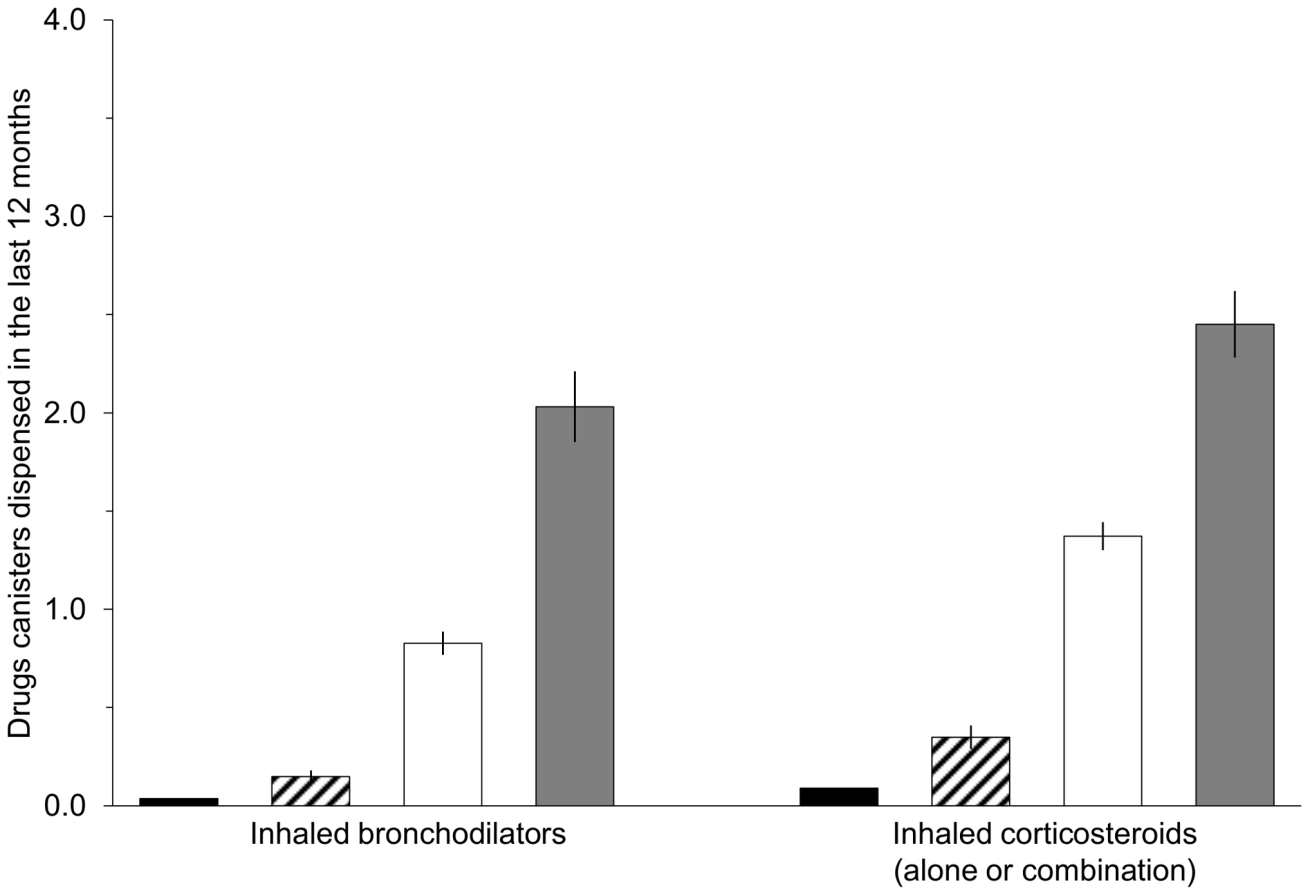
Asthma drugs dispensed in the “Inconsistent answers” pattern. The figures represent the means +/− standard error of canisters dispensed during the 12 months preceding the exact return date of the 2005 questionnaire. Black box: “Never asthma” pattern (taken as the descriptive reference, n = 64,061); Among women from the “Inconsistent” pattern with no missing answer to the ever asthma question in 2005 (n = 2,954): Striped box: “Inconsistent” pattern with a negative answer to ever asthma in 2005 (n = 659); White box: “Inconsistent” pattern with a positive answer to ever asthma in 2005, and with no asthma attack in the past 12 months (n = 1,746); Grey box: “Inconsistent” pattern with a positive answer to ever asthma in 2005, and with asthma attack in the past 12 months (n = 417). Women with a positive answer to the ever asthma question in 2005 but with missing data on asthma attack in the past 12 months were not included (n = 132). All differences were statistically significant at the p<0.001 level.

Figure 2 shows the association of reported attacks in the last 12 months with drugs dispensed during the same period, in women from the “Inconsistent” pattern. In women who reported ever asthma in the 2005 questionnaire, ICS and inhaled bronchodilators were 1.78 and 2.44 times more frequently dispensed to women who reported an attack in the last 12 months compared to women who did not. Among women who did not report ever asthma in the 2005 questionnaire, the asthma drugs dispensed were markedly lower than in those who reported ever asthma. However, they were 3.89 times higher for ICS and 3.75 times higher for inhaled bronchodilators than for women from the “Never asthma” pattern (taken as the descriptive reference). The number of inhaled bronchodilator canisters dispensed to women with an attack in the last 12 months was similar comparing “Inconsistent” and “Consistent” patterns (mean (SD): 2.03 (3.67) vs. 2.38 (3.87) respectively; p = 0.14).

**Figure 3:**
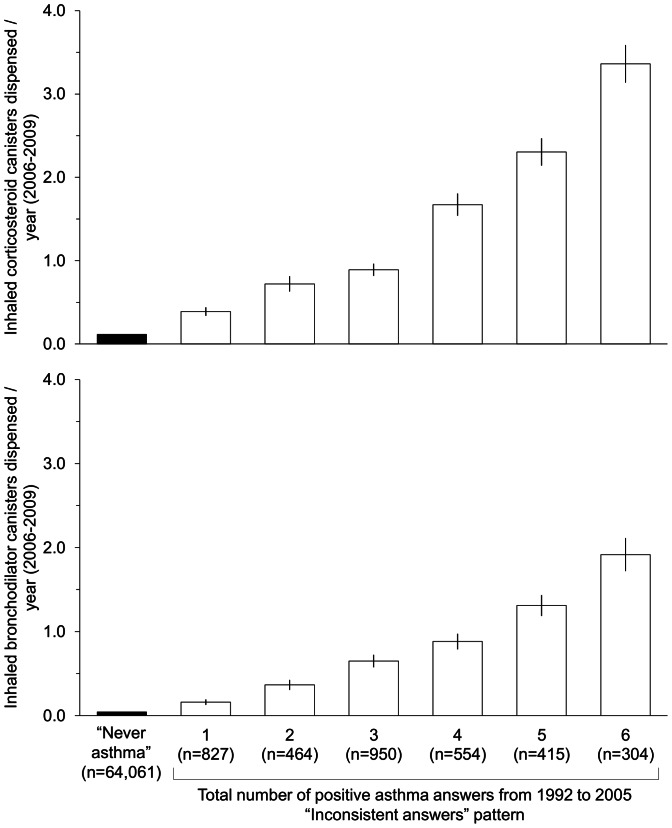
Asthma drugs dispensed and total number of positive asthma answers in the “Inconsistent answers” pattern. The figures represent the means +/− standard error of canisters dispensed during the 4-year period 2006–2009. The “Never asthma” pattern was taken as the descriptive reference. All p for trend were <0.001.

The “Inconsistent answers” pattern was further studied. Figure 3 shows the increase in asthma drugs dispensed during the 2006–2009 period with the total number of positive answers to the asthma question between 1992 and 2005 (from 1 to 6). The annual mean dispensed canisters ranged for ICS from 0.39 for only one positive answer to 3.36 for six positive answers; and for inhaled bronchodilators, from 0.16 to 1.92 (p for trend <0.001). The proportion of women with regular ICS medication every year increased with the number of positive answers to the asthma question (3.1%, 6.9%, 8.4%, 16.6%, 24.8% and 37.5% for women with one isolated positive answer, 2, 3, 4, 5 and 6 positive answers, respectively). Reports of asthma attacks in the last 12 months both in the 2002 and 2005 questionnaires increased with the number of positive answers to ever asthma between 1992 and 2005 (in 2002∶7.5%, 10.3%, 17.2%, 24.9% and 38.0% for those with one or two, 3, 4, 5 and 6 positive answers; similarly in 2005∶8.2%, 12.4%, 16.3%, 28.2% and 34.3% for one or two, 3, 4, 5 and 6 positive answers respectively).

Due to the potential overlap between asthma and COPD in old-age, a sensitivity analysis was conducted among never smokers (n = 38,286 from “Never asthma” pattern, n = 1,993 from “Inconsistent” pattern, n = 1,581 from “Consistent” pattern). The analysis led to similar results. Moreover, the average number of anticholinergic canisters dispensed in the three patterns was close to 0 each year (2004–2009 annual mean (SD): 0.01 (0.22) in the “Never asthma” pattern, 0.08 (0.62) in the “Inconsistent” pattern, 0.16 (0.84) in the “Consistent” pattern).

## Discussion

In this large population, we defined three temporal asthma patterns using seven repeated answers to the question “Have you ever had an asthma attack?”. The sound reliability of the patterns has been confirmed, considering the questionnaires as well as the reimbursement of asthma drugs. Three or more repeated positive asthma answers from 1992 to 2005 ensured an almost perfect agreement with the standardized asthma definitions. We have shown that asthma patterns with “Inconsistent answers” and “Consistent answers” corresponded to asthma with different characteristics regarding age of onset and activity. As women with less severe, more variable asthma are more likely to be inconsistent in their responses, we therefore hypothesize that the pattern labeled “Inconsistent answers” is a phenotype which may capture the variable expression of asthma. Furthermore, the repetition of positive answers is an indicator of disease severity.

As an accepted “objective” definition of asthma does not exist, and without available lung function tests, standardized definitions and reimbursement data were taken as a reference to assess the reliability of the asthma patterns. Studies have shown the validity and reliability of the standardized definitions of asthma [16] and the feasibility of using reimbursement databases in asthma [6]. All three asthma patterns corresponded to three distinct consumptions of asthma drugs with very similar values over 6 years, showing coherence and stability over time and a sound internal consistency of the data.

Results show that the assessment of ever asthma is reinforced with repeated questionnaires. Regardless of the order and of their position over time, three positive answers provided a very high concordance with standardized definitions, an observation which could more often help to study asthma in large general health surveys.

The two asthma patterns (“Consistent answers” and “Inconsistent answers”) shared very high proportions of women with the BMRC and ATS definitions of asthma. In ECRHS, the analysis of inconsistent answers regarding the question “Have you ever had asthma?” was conducted considering Survey 2 compared to Survey 1, carried out 10 years earlier. Pattaro et al. showed that those with inconsistent answers reported more asthma-like symptoms and use of medicines, and had more bronchial hyperreactivity - an objective measure - as compared to participants with no positive answers [17]. Unfortunately, spirometry or bronchial challenge tests were not available in the E3N population.

Several repeated questionnaires are of great interest, as the “Inconsistent answers” pattern seemed to group women with variable disease. Answers to the asthma question were associated with the drugs dispensed (controller and reliever) in the past year, suggesting that the answers relate to the recent course of the disease. Part of the answer variability in the “Inconsistent” pattern could be explained by asthma varying in activity and severity, with intermittent symptoms and disease-free intervals. Besides, Torén et al. have already shown that the question on ever asthma (“Do you have or have you ever had asthma?”) was dependent on disease severity, estimated by the minimum amount of medication required to maintain the control of asthma [18]. Intermittent asthma could also explain the lower amount of asthma drugs dispensed in the “Inconsistent” (variable) pattern compared to “Consistent” by an irregular treatment related to irregular symptoms.

The other part of the answer variability is probably related to remitted asthma and recall bias, as women from the “Inconsistent” pattern reported childhood asthma more frequently than women from the “Consistent” pattern. Although studies with follow-up since childhood have evidenced recall bias regarding childhood asthma assessed retrospectively in childhood [19], the validity of the reported age of asthma onset is considered as reasonably accurate [17], [18]. In the E3N study, an analysis comparing ages of onset declared in 2002 and 2005 showed very good agreement (kappa [95% confidence interval]: 0.92 [0.91–0.94]). Furthermore, among the subsample of women included in the detailed respiratory health survey in 2009, 93% confirm the age of onset declared in one of the previous questionnaires. In addition, the expected protective effect of farming exposure on childhood asthma, assessed through the reported age of childhood onset, has been evidenced in the E3N study [9], supporting the accuracy of the reported age of onset.

Importantly, the repetition of positive answers over time could be a marker of disease severity, independently of the order. The number of women requiring regular controller medication each year was 10 times higher when considering 6 positive answers compared to one isolated positive answer. The number of dispensed inhaled bronchodilators also increased when the number of positive answers increased. Women with at least 3 positive answers from 1992 to 2005 had had more asthma attacks in the past 12 months, and more asthma drugs dispensed, suggesting difficult-to-control and severe asthma. As these drugs need a doctor’s prescription, repeated treatment ensured the regular presence of a doctor, supporting the idea of severe asthma requiring repeated visits.

Limitations of this study include the potential misclassification between asthma and COPD in old-age. Self-reported asthma might have included COPD. It is unlikely that there were numerous cases of COPD as most of the women were never and former smokers, and the number of anticholinergic canisters dispensed each year was close to 0. Results restricted to never smokers remained similar. Another limitation concerns the generalization of the present findings obtained in women covered by a national health insurance. Caution is required regarding generalization, in particular to men and in populations with reduced health care access. Females from the French E3N study are more health-conscious than the general population, as they consist of teachers who have an intermediate to advanced level of education. The E3N population was fairly representative however of the whole population insured by the health insurance plan [7].

In conclusion, understanding the variable expression of asthma is a major scientific question, at the clinical and epidemiological level. We have shown that studies on variable asthma, for which there are no standards as yet, may be conducted based on the variability of the responses, even to such a simple question as “ever asthma”. The availability of comprehensive dispensed drugs has provided new insight into the understanding of the clinical variability of asthma. Research is needed in various populations to understand the phenotypic characteristics of this variable pattern, to assess its determinants (considering environmental and genetic factors as well as suboptimal treatment) and to evaluate its predictive value regarding exacerbations, in order to improve its clinical management.

## Supporting Information

File S1 **In this Supporting Information, we have expanded some of the methods, particularly those regarding the setting.** We also present a detailed analysis of the missing data, as well as the analysis of reliability of temporal asthma patterns. This analysis of reliability was based on a respiratory health survey among a random sample of the population, and on the dispensed drug database.
(DOC)
