# Supplementary material for: Temporal Asthma Patterns Using Repeated Questionnaires over 13 Years in a Large French Cohort of Women
Source: PLoS One. 2013 May 31;8(5):e65090. doi: 10.1371/journal.pone.0065090 (PMC3669014; doi:10.1371/journal.pone.0065090)

**File S1**

**Supporting Information**

**Temporal asthma patterns using repeated questionnaires over 13 years in a large French cohort of women**

Margaux Sanchez, Jean Bousquet, Nicole Le Moual, Bénédicte Jacquemin, Françoise Clavel-Chapelon, Marc Humbert, Francine Kauffmann, Pascale Tubert-Bitter, Raphaëlle Varraso

In this Supporting Information, we have expanded some of the methods, particularly those regarding the setting. We also present a detailed analysis of the missing data, as well as the analysis of reliability of temporal asthma patterns. This analysis of reliability was based on a respiratory health survey among a random sample of the population, and on the dispensed drug database.

**Materials and methods**

Study population

The E3N study is a prospective study of major chronic diseases among members of a French national health insurance plan covering mostly teachers. Briefly, in 1990, 500,000 women aged 40–65 years, living in continental France, and all of whom were members of the Mutuelle Générale de l’Education Nationale (MGEN) were invited to participate. 20% of these women agreed to participate by filling in the first questionnaire (1990) and the consent form (n=98,995). The females included were fairly representative of the population covered by the health insurance plan regarding age and geographic region. The general characteristics of this population have been reported elsewhere [1]. Part of the E3N cohort is also included in the European Prospective Investigation on Cancer [2]. The 2^nd^ (1992), 3^rd^ (1993) and 4^th^ (1995) questionnaires were sent only to the women who had answered the previous questionnaire (Figure S1). As from the 5^th^ questionnaire (1997), questionnaires were sent to all of the women who had responded to the first and who had not asked to be withdrawn from the study. Our study population was composed of 70,428 women who returned the 8^th^ questionnaire in 2005 and who were still alive in 2009. No pulmonary function tests were performed. Questionnaires were self-completed and returned by mail.

Setting

Main E3N study – Using the seven repeated answers to the question “Have you ever had an asthma attack?” between 1992 and 2005, three temporal asthma patterns were defined. The first pattern included all women never having responded “yes” to the asthma question between 1992 and 2005. This temporal pattern was labeled “Never asthma” owing to the absence of positive answers over time. The second pattern included all women who responded “yes” at least once, followed by a “no” in a subsequent questionnaire. This pattern was labeled “Inconsistent answers”. The third pattern included all women who responded “yes” at least once, with consistency in all following questionnaires (i.e. never “no”). This pattern was labeled “Consistent answers”.

Respiratory health survey - Among the 70,428 women of the study population (those who returned the 8^th^ questionnaire in 2005 and were still alive in 2009), 48,498 answered to the asthma question in 2005 (Figure S1). Among these women, we selected 260 in each temporal pattern (780 women). In addition, 260 women with a missing answer to the asthma question in 2005 were selected, regardless of their previous answers. Each of the four groups included 200 women selected at random, as well as 60 women randomly selected among current smokers. In 2009, all of these 1,040 women received a detailed questionnaire based on international standardized recommendations to better characterize asthma with adapted questions from The British Medical Research Council (BMRC) [3], the American Thoracic Society and the Division of Lung Diseases (ATS) [4] and the ECRHS [5]. The response rate was high (92.3%) with 960 questionnaires returned. Using this detailed questionnaire, we were able to investigate the British Medical Research Council (BMRC) asthma definition (asthma attack or attack of shortness of breath at rest with wheezing), the ATS asthma definition (doctor diagnosis of asthma), the asthma symptom score in the last 12 months proposed by Pekkanen et al. [6, 7] (number of positive responses to 5 asthma symptoms), as well as adult-onset asthma (age at first attack >16 years).

Dispensed drug database - Ethical approval was granted to use the database for this research. Using the Anatomical Therapeutic Chemical (ATC) classification [8], we identified all dispensed asthma medications. In particular, we considered inhaled corticosteroids (ICS) alone or combination (ATC codes R03BA02, R03BA03, R03BA05, R03AK06 and R03AK07) and inhaled bronchodilators (ATC codes R03AC02, R03AC03, R03AC08, R03AC12, R03AC13, R03CC03, R03AK03 and R03AK04) [9]. Inhaled corticosteroids and inhaled bronchodilators were always considered separately.

**Results**

Missing data on ever asthma in 2005 questionnaire

The 21,930 women with missing data in the asthma answer in 2005 were slightly (+0.6 years) but significantly (p<0.001) older than the 48,498 women with no missing data (Table S1). Women with a missing asthma answer were less educated (15.3% vs. 10.3% with <11 years of education) and more often never smokers as compared to women with no missing answer. No difference was found regarding body mass index. Results were in agreement with current knowledge, assuming that the women with a lower education level had answered less to questions.

Among the women with a missing answer on asthma in 2005, 95.8% (n=20,998) belonged to the “Never asthma” pattern (as compared to 88.8% among women without a missing answer) showing that a missing response on asthma in 2005 corresponded in most of the cases to women without previous positive asthma answers. In the subsample of women surveyed in 2009 with a specific respiratory health questionnaire, only 5.0% answered positively to the ever asthma question (n=12; n=230 with negative answers).

In the “Never asthma” pattern, women with missing data on asthma in 2005 had significantly higher reimbursements of ICS and inhaled bronchodilators (respectively +0.04 and +0.01) than women with no missing data. This statistical difference was due to the large population and was not clinically meaningful.

Reliability of the three temporal patterns (using the respiratory health survey in 2009).

Almost all of the women from the “Never asthma” pattern answered negatively to the ever asthma attack question in 2009 (96.7%), and two-thirds (67.6%) did not declare any of the 5 symptoms from the asthma symptom score (Table S2). Unexpectedly, 29.2% declared an attack of shortness of breath after exercise in the last 12 months, while the four other symptoms were declared by less than 8%. No differences were found between “Inconsistent” and “Consistent” patterns with regard to ATS and BMRC asthma definitions (p=0.37, p=0.47 respectively), ever asthma drug taking (p=0.64), severe asthma exacerbations (hospitalizations (p=0.86), emergency room (p=0.84), night in hospital (p=0.52)), and the asthma symptom score (≥ 2 symptoms were reported by 30.7% vs. 35.1%, p=0.20, respectively “Inconsistent answers” and “Consistent answers” patterns).

Agreement between temporal asthma patterns with standardized asthma definitions and adult-onset asthma was defined as substantial (from 0.75 to 0.80 for the “Inconsistent” pattern, from 0.72 to 0.77 for the “Consistent” pattern) (Table S3). For the asthma symptom score >2, agreement was poor (0.23 for the “Inconsistent” pattern, 0.28 for the “Consistent” pattern).

The agreement between temporal asthma patterns and asthma definitions increased with the total number of positive asthma answers between 1992 and 2005. In the “Consistent” pattern, agreement ranged from 0.62 for an isolated positive answer to 0.92 for 3 or more positive answers using the ATS definition as a gold standard. In the “Inconsistent” pattern, the increase was greater, as each additional positive answer led to an increase of at least 0.21 in the agreement. Three or more positive answers ensured an almost perfect agreement. Very good agreements with international definitions of asthma (ATS and BMRC) suggest the predictive role of the temporal patterns regarding women with asthma. Conversely, asthma symptom score as a reference definition led to a very low agreement, probably due to the high proportion of “Never asthma” declaring an attack of shortness of breath after exercise. This question could quite easily be misunderstood, especially in an elderly population.

The average number of asthma drugs dispensed in the “Never asthma” pattern was close to 0 each year (2004-2009 annual mean (SD), ICS: 0.10 (0.58), inhaled bronchodilators: 0.01 (0.43)). Women with “Inconsistent answers” were reimbursed two times less than those with “Consistent answers” (2004-2009 annual mean (SD), ICS: 1.23 (2.63) vs. 2.24 (3.39); inhaled bronchodilators: 0.73 (2.09) vs. 1.27 (2.83), “Inconsistent” and “Consistent” patterns respectively). The average number of dispensed ICS was constant throughout the period.

**References**

1. Clavel-Chapelon F, van Liere MJ, Giubout C, Niravong MY, Goulard H, et al. (1997) E3N, a French cohort study on cancer risk factors. E3N Group. Etude Epidémiologique auprès de femmes de l'Education Nationale. Eur J Cancer Prev 6: 473-478.
2. Riboli E (1992) Nutrition and cancer: background and rationale of the European Prospective Investigation into Cancer and Nutrition (EPIC). Ann Oncol 3: 783-791.
3. British Medical Research Council/Communauté Européenne du Charbon et de l'Acier. Brille D, Casula D, van der Lende R, Smidt U, Minette A rapp (1967) Commentaires relatifs au questionnaire pour l'étude de la bronchite chronique et de l'emphysème pulmonaire. Luxembourg : CEE-CECA, Collection d'hygiène et de médecine du travail, n°14, 1971.
4. Ferris BG (1978) Epidemiology Standardization Project (American Thoracic Society). Am Rev Respir Dis 118: 1-120.
5. Burney PG, Luczynska C, Chinn S, Jarvis D (1994) The European Community Respiratory Health Survey. Eur Respir J 7: 954-960.
6. Pekkanen J, Sunyer J, Anto JM, Burney P (2005) Operational definitions of asthma in studies on its aetiology. Eur Respir J 26: 28-35.
7. Sunyer J, Pekkanen J, Garcia-Esteban R, Svanes C, Künzli N, et al. (2007) Asthma score: predictive ability and risk factors. Allergy 62: 142-148.
8. WHO Collaborating Centre for Drug Statistics Methodology (2011) Guidelines for ATC classification and DDD assignment. Oslo, 2010. Available: http://www.whocc.no/filearchive/publications/2011guidelines.pdf. Accessed: April 30 2013.
9. Furu K, Karlstad Ø, Skurtveit S, Håberg SE, Nafstad P, et al. (2011) High validity of mother-reported use of antiasthmatics among children: a comparison with a population-based prescription database. J Clin Epidemiol 64: 878-884.
10. Landis JR, Koch GG (1977) The measurement of observer agreement for categorical data. Biometrics 33: 159–174.

**Table S1.** Characteristics and asthma drugs dispensed according to missing/no missing answer to ever asthma question in 2005.

|  | Missing answer to ever asthma question in 2005 | No missing answer to ever asthma question in 2005 | p-value |
| --- | --- | --- | --- |
| **Questionnaire returned in 2005, n** | **21,930** | **48,498** |  |
| Age in 2005 (years), mean (SD) | 64.9 (6.4) | 64.3 (6.4) | ** |
| Smoking habits in 2005, % |  |  |  |
| Never smokers | 62.5 | 60.1 | ** |
| Former smokers | 31.4 | 33.5 |
| Current smokers | 6.1 | 6.4 |
| Body mass index in 2005 (kg/m²), mean (SD) | 23.9 (3.8) | 24.0 (3.8) | 0.13 |
| Education number of school years, % |  |  |  |
| ≤ 11 years | 15.3 | 10.7 | ** |
| 12 to 14 | 51.4 | 51.0 |
| 15 to 16 | 16.8 | 19.5 |
| ≥ 17 | 16.5 | 18.8 |
| Temporal patterns, % |  |  |  |
| “Never asthma” | 95.8 | 88.8 | ** |
| “Inconsistent answers” | 2.5 | 6.1 |
| “Consistent answers” | 1.7 | 5.1 |
| **In “Never asthma” pattern only, n** | **20,998** | **43,063** |  |
| Canisters dispensed /year 2004-2009, mean (SD), min-max |  |  |  |
| Inhaled corticosteroids (alone or combination) | 0.13 (0.70), 0-13 | 0.09 (0.52), 0-16 | ** |
| Inhaled bronchodilators | 0.05 (0.48), 0-18 | 0.04 (0.40), 0-31 | ** |

p-value from χ² test for categorical variable and from student test for continuous variable

*p<0.05, ** p<0.001

**Table S2.** Description of asthma according to 1992-2005 temporal asthma patterns.

|  | **1992-2005 temporal asthma patterns** | | |
| --- | --- | --- | --- |
|  | “Never  asthma” | “Inconsistent  answers” | “Consistent  answers” |
| **Respiratory questionnaire in 2009, n** | **242** | **238** | **233** |
| Ever had an asthma attack |  |  |  |
| No | 96.7 | 17.6 | 21.9 |
| Yes | 1.2 | 77.7 | 73.8 |
| Missing data | 2.1 | 4.6 | 4.3 |
| *Among women with no missing data on asthma, n* | *237* | *227* | *223* |
| Asthma doctor diagnosis § | 0.8 | 74.0 | 70.9 |
| Age at first asthma attack >16 years | 100.0 | 52.0 | 54.3 |
| Asthma attack last 12 months | 0.8 | 16.7 | 22.0* |
| Ever had an attack of shortness of breath at rest with wheezing | 1.7 | 23.5 | 20.2 |
| Ever had an asthma attack or an attack of shortness of breath at rest with wheezing † | 2.5 | 79.4 | 74.7 |
| Taking drugs for asthma (ever) |  | 68.1 | 64.8 |
| Hospitalization for asthma (ever) |  | 8.0 | 7.7 |
| Night in hospital for asthma (ever) |  | 7.6 | 5.2 |
| Emergency room for asthma (ever) |  | 7.1 | 8.6 |
| *Among women with no missing data on symptoms in the last 12 months, n* | *216* | *199* | *188* |
| Breathless while wheezing | 5.1 | 26.6 | 30.9 |
| Woken up with a feeling of chest tightness | 7.9 | 23.1 | 30.9 |
| Attack of shortness of breath at rest | 3.2 | 15.1 | 14.9 |
| Attack of shortness of breath after exercise | 29.2 | 44.7 | 55.3* |
| Woken by an attack of shortness of breath | 1.9 | 9.0 | 10.6 |
| Asthma symptom score || |  |  |  |
| 0 | 67.6 | 41.7 | 33.5 |
| 1 | 24.1 | 27.6 | 31.4 |
| 2 | 5.1 | 14.1 | 10.6 |
| 3 | 1.4 | 6.5 | 12.8 |
| 4 | 0.5 | 7.0 | 6.9 |
| 5 | 1.4 | 3.0 | 4.8 |

Data presented as %, unless otherwise stated. Respiratory questionnaires were sent to a subsample of the E3N population in 2009.

*p<0.05 **p<0.001 from χ² test “Inconsistent” pattern vs. “Consistent” pattern;

§ American Thoracic Society definition of asthma;

† British Medical Research Council definition of asthma;

|| Number of symptoms in the last 12 months.

**Table S3.** Agreement between 1992-2005 temporal asthma patterns with standardized asthma definitions.

|  | **1992-2005 temporal asthma patterns** | |
| --- | --- | --- |
|  | “Inconsistent  answers” | “Consistent  answers” |
| **Respiratory questionnaire in 2009, n** | **238 *** | **233 †** |
| Ever had an asthma attack or an attack of shortness of breath at rest with wheezing ‡ |  |  |
| All women | 0.80 | 0.77 |
| Total number of positive ever asthma attack answers from 1992 to 2005: |  |  |
| 1 | 0.41 | 0.68 |
| 2 | 0.70 | 0.80 |
| >3 | 0.93 | 0.91 |
| Asthma diagnosed by a doctor § |  |  |
| All women | 0.76 | 0.72 |
| Total number of positive ever asthma attack answers from 1992 to 2005: |  |  |
| 1 | 0.31 | 0.62 |
| 2 | 0.70 | 0.79 |
| >3 | 0.91 | 0.92 |
| Adult asthma |  |  |
| All women | 0.75 | 0.72 |
| Total number of positive ever asthma attack answers from 1992 to 2005: |  |  |
| 1 | 0.33 | 0.62 |
| 2 | 0.62 | 0.73 |
| >3 | 0.92 | 0.92 |
| Asthma symptom score >2 || |  |  |
| All women | 0.23 | 0.28 |
| Total number of positive ever asthma attack answers from 1992 to 2005: |  |  |
| 1 | 0.16 | 0.25 |
| 2 | 0.18 | 0.07 |
| >3 | 0.27 | 0.57 |

Data presented as Kappa, calculated using the “Never asthma” temporal pattern as reference. Classification system developed by Landis and Koch [10]: κ<0.40 indicates poor agreement, 0.41-0.60 moderate agreement, 0.61–0.80 substantial agreement and values of 0.81 to 1.00 indicate almost perfect to perfect agreement.

* 238 women of whom 17%, 11% and 72% had respectively 1, 2 and >3 positive answers from 1992 to 2005;

† 233 women of whom 38%, 31% and 31% had respectively 1, 2 and >3 positive answers from 1992 to 2005;

‡ British Medical Research Council definition of asthma;

§ American Thoracic Society definition of asthma;

|| Number of symptoms in the last 12 months: breathless while wheezing, woken up with a feeling of chest tightness, attack of shortness of breath at rest, attack of shortness of breath after exercise, woken by attack of shortness of breath.

**Figure S1.** Flow diagram of the E3N cohort study and respiratory health survey.


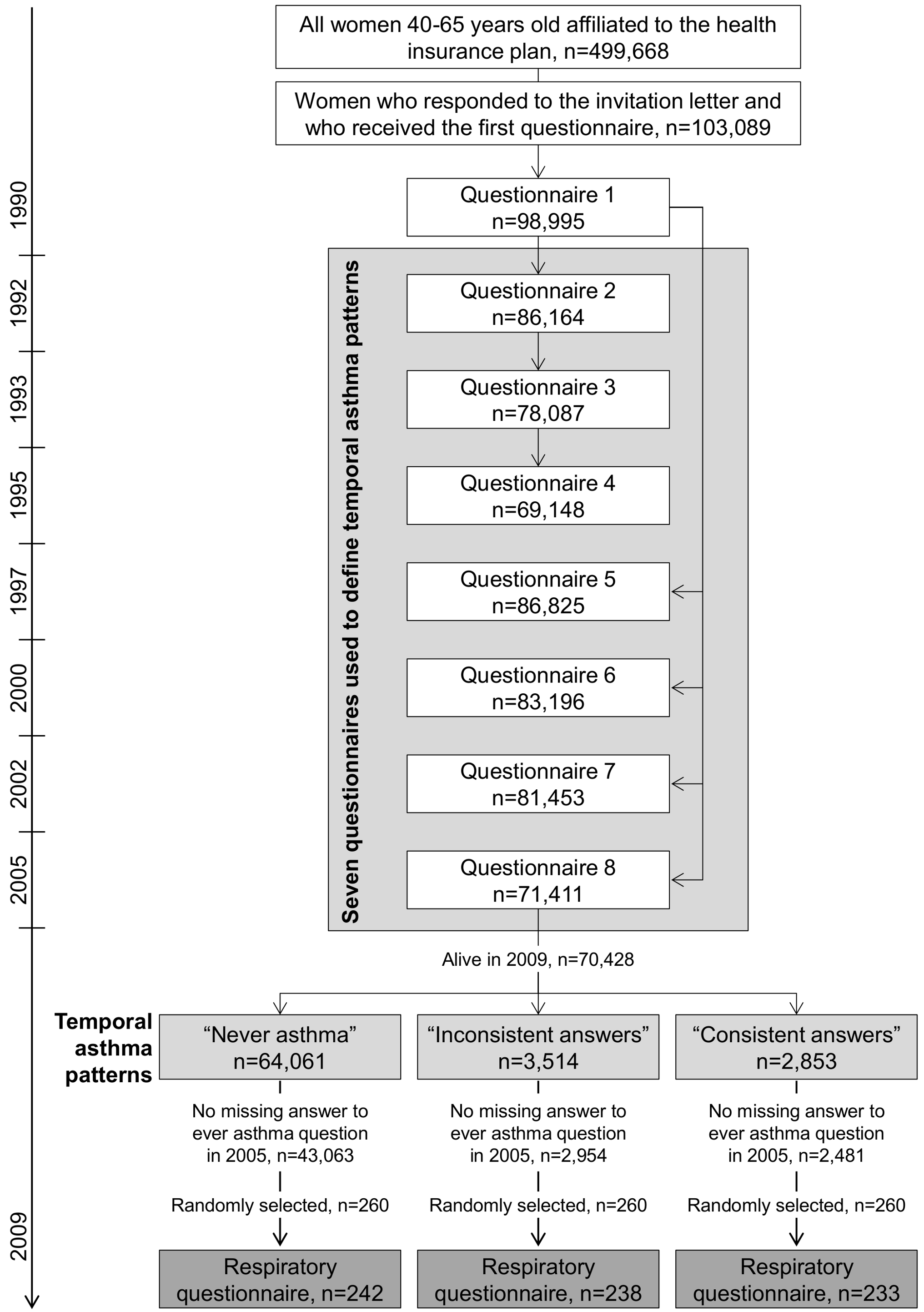

Supplement: File S1 — In this Supporting Information, we have expanded some of the methods, particularly those regarding the setting. We also present a detailed analysis of the missing data, as well as the analysis of reliability of temporal asthma patterns. This analysis of reliability was based on a respiratory health survey among a random sample of the population, and on the dispensed drug database. (DOC) [file pone.0065090.s001.doc]
